# A Preliminary Investigation of Reversing RML: From an RDF dataset to its Column-Based data source

**DOI:** 10.3897/BDJ.3.e5464

**Published:** 2015-07-29

**Authors:** Carlo Allocca, Alexandros Gougousis

**Affiliations:** ‡ Hellenic Center for Marine Recearch (HCMR), Institute of Marine Biology, Biothechnology and Aquaculture (IMBBC), Thalassokosmos, 71003, Heraklion, Crete, Greece

**Keywords:** Reverse Mapping Language, RML, Linked Open Data, Data Preservation

## Abstract

**Background:**

A large percentage of scientific data with tabular structure are published on the Web of Data as interlinked RDF datasets. When we come to the issue of long-term preservation of such RDF-based digital objects, it is important to provide full support for reusing them in the future. In particular, it should include means for both players who have no familiarity with RDF data model and, at the same time, who by working only with the native format of the data still provide sufficient information. To achieve this, we need mechanisms to bring the data back to their original format and structure.

**New information:**

In this paper, we investigate how to perform the reverse process for column-based data sources. In particular, we devise an algorithm, RML2CSV, and exemplify its implementation in transforming an RDF dataset into its CSV tabular structure, through the use of the same RML mapping document that was used to generate the set of RDF triples. Through a set of content-based criteria, we attempt a comparative evaluation to measure the similarity between the rebuilt CSV and the original one. The results are promising and show that, under certain assumptions, RML2CSV reconstructs the same data with the same structure, offering more advanced digital preservation services.

## Introduction

To date, a large percentage of scientific data published on the Web of Data (Bizer et al. 2009) comes from tabular source (Tennison and Kellogg 2015), commonly made available in the format of comma separated values (CSV). When those contents need to be exposed to the Web following the Linked Open Data principles (Heath and Bizer 2011), they are usually transformed to interlinked RDF datasets (Tzitzikas et al. 2013). Accordingly, a major issue related to the long-term preservation (Shaon et al. 2012, Stefanova and Risch 2013a, Tzitzikas et al. 2012) of such RDF-based digital objects is the ability to provide full support for their reuse, including means for those users who have no familiarity with RDF data model, and, at the same time, work with the original tabular format of the data. The latter is a very common format to work with (Kaschner et al. 2008) for scientists including biologists, geologists and so forth, and it is often sufficient to build scientific models and test/validate their hypothesis (Candela et al. 2013). For such cases, the reuse of preserved RDF datasets would require a heavy ad-hoc pre-processing for *understanding* (Flouris and Meghini 2007), *extracting* and *arranging* (Stefanova and Risch 2013a) the data that satisfy the user intended use, including the transformation of the RDF data back to their original format (Stefanova and Risch 2013b). While several approaches have been proposed to map different types of data sources to RDF data model (R2RML (Das et al. 2012), RML (Dimou et al. 2013), X3ML (Kondylakis et al. 2006), just to mention a few), the opposite direction, that is transforming an RDF dataset back to its original data source has not been yet attempted to our knowledge, especially for the case of column-based sources. In this paper, we investigate the reverse process that performs the reconstruction of the original data source from an RDF dataset. We devise a generic and extendable algorithm, notably the RML2CSV, and exemplify the computing of the process for its automatic implementation. In contrast with the approaches described in the *Related Works* section, RML2CSV aims to rebuild a CSV data source that reflects *not any* but the same column-based structure and content of the original data source. To achieve this, the proposed method is based on RML (Dimou et al. 2013), in the sense that it makes use of the same RML Mapping Document - set of mapping rules - that was used to generate the RDF dataset.

Based on a set of content-based criteria to measure the similarity between the original data source and the one reconstructed by RML2CSV, we evaluate the approach over a collection of real-world RDF datasets from Biodiversity domain available in the MedObis repository (Arvanitidis et al. 2006). The results demonstrate the feasibility of the reverse process under certain assumptions that are more related to the nature of the original data source and the “quality” of the mapping rules rather than related to the algorithm *per se*. RML2CSV rebuilds the content with the data structure as the original one, offering more advanced digital preservation services in supporting long-term access.

The paper continues as in the following: The *Study Area Description* section briefly describes the underlying R2RML and RML mapping languages, demonstrating how they work in practice for exposing a CSV data source as an RDF dataset, and introduces the reverse process. It also details the main assumptions under which we analyse and develop the reverse process. The *Design Description and Implementation* sections describe the RML2CSV algorithm and its implementation. The *Evaluation and Results* section defines the main criteria to evaluate the approach and details the results. The *Discussion* section discusses upon the achievements and propose a number of solutions for relaxing the two assumptions that we will be part of future development. The *Related Works* section discusses relevant works. Finally, *Conclusion and Outlook* section concludes the work describing the main achievements and provide a road-map for future work.

## Project description

### Study area description

First, we briefly introduce the R2RML and RML ([R2]RML) mapping languages to the extent at which it concerns with our preliminary investigation (see (Das et al. 2012) and (Dimou et al. 2013) for a thorough knowledge and complete details of both languages). Then, we describe an example of using RML for both the forward and reverse processes. Finally, we set the main assumptions under which we analyze the reverse problem.

**An R2RML and RML Overview.** R2RML provides a declarative language for expressing customized mappings from relational database to RDF dataset, expressed in a structure and target vocabulary of the Engineer's mapping choice (Das et al. 2012). Based on the same principle, RML provides an extension for expressing customized mapping rules from heterogeneous data structures and serializations (such as CSV, XML JSON) to the RDF data model (Dimou et al. 2013). The set of mapping rules is provided as an *R2[RML] Mapping Document*. It is any document written in the Turtle RDF syntax that encode an [R2]RML *mapping*. The latter is a structure that consists of one or more *triples maps* that specify the rules for translating, for the case of a CSV data source, each record to zero or more RDF triples. Specifically, a *triples map* is represented by a resource that: (1) it must have exactly one *logical source* that points to the data source (of type CSV) that contains the data to be mapped to RDF triples; (2) it must have exactly one *subject map* that specifies how to generate a subject for each record of the logical source; and (3) it may have zero or more *predicate-object-maps* that specifies pairs of predicate maps and object maps that, together with the subject generated by the subject map, may form one or more RDF triples for each record of the (CSV) data source (Das et al. 2012).

To face with the high expressivity of RML's mapping language and to monitor the complexity of the *reverse process*, we have finalised, implementation included, the current work considering a subset of RML: RML Lite. The main restrictions that RML Lite imposes to a triples map are:

1. given a mapping rule tm_i_, the *subject map* is characterized by one template property and one class property with IRIs values,

2. given a mapping rule tm_i_, the *predicate-object-Map* references to one *predicate property* with IRI value and one *object map* which, in turn, is represented by an *objectMap property* with one value of *referencing object map* type,

3. given a mapping rule tm_i_, a *referencing object map* is represented by a resource that has exactly one *parentTriplesMap property* where the value must be a triples maps as defined above, known as the referencing object map's parent triples map,

4. if tm_1_,...,tm_n_ are triples maps of the same RML Mapping Document and defined according to 1-3, they all refer to the same CSV data source.

**Figure 1.**
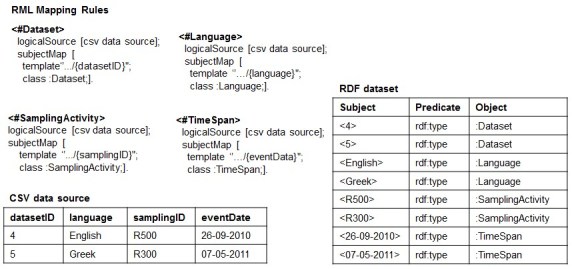
An example of CSV data source exposed as an RDF dataset using a set of RML rules.

Basically, RML Lite allows only the mapping of CSV *columns* to *Class* or *Object Property* of an RDF data model and, at the same time, it is expressive enough to discuss potential issues related to the *reverse process* in general, and how we intend to approach them. An example of RML Lite Mapping document is showed in Fig. 1.

**The CSV2RDF and RDF2CSV Processes.** Generally speaking, mapping process aims at transforming instances of a data source structure into instances of target schema, preserving the semantic and allowing the implementation of an automatic algorithm to perform such a transformation (Kondylakis et al. 2006).

When using RML to perform such a task for a CSV data source (CSV2RDF), it means to write down a set of rules (stored as [R2]RML *mapping document*) that specifies how to semantically interpret both the structure and the data with respect to the target RDF data model. For example, Fig. 1 shows an example of RML mapping document with four rules: <#Dataset>, <#Language>, <#SamplingActivity> and <#TimeSpan>, where each rule declares how to transform the corresponding column and its values into RDF triples. For instance, the rule <#Dataset>, when applied, transforms the values of column datasetID into instances of the class: Dataset, e.g. <4 rdf:type: Dataset> and <4 rdf:type: Dataset>.

Conversely, RDF2CSV - the task of rebuilding the structure and the instances of the CSV data source from the RDF dataset - works in opposite direction: the RML rules are used to rebuild the column-based structure and populate it with the data from the RDF dataset. To exemplify, the rule <#Dataset>, when applied for the reverse process, retransforms the instances of the class: Dataset into values of the column datasetID.

**Assumptions.** The RDF2CSV process can pose a number of issues making it a very challenging task to accomplish. In what follow we present and discuss two of them: the first is related to the set of RML mapping rules used to expose the CSV data source in RDF and, the second concerns with the *implicit* cardinality constraints of the associations between the columns of the CSV data source. For both, in this preliminary study, we formulate assumptions to work with.

**Figure 2.**
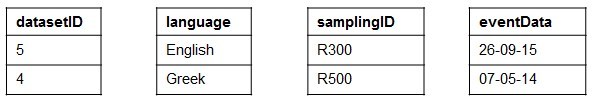
An example of output when reversing independent mappings.

***1. The Dependency Tree Assumption*:** It is related to the implicit structure that the set of RML mapping rules should form in order to succeed with the reverse process. Before formalizing it, we explain it by continuing the reverse of the RDF dataset of Fig. 1. Specifically, the rule <#Language> tell us that the instances of the class: Language are the values of the column language. Likewise, the rules <#SamplingActivity> and <#TimeSpan> would produce structure and data for the samplingID and the eventData columns, respectively. The result is showed in Fig. 2.

What we have produced so far are only two dimensions (the columns and the cells) out of the three (the columns, the cells and the rows) that characterize a CSV data model. Tennison and Kellogg 2015 defines a CSV in such a way that, for each row, the associated cells are (implicitly) kept together by including them in the same line. This is not the case for the RDF data model. Actually, the corresponding RDF triples may not be connected practically and, the RDF data model does not keep any specific order or relationship between them (Stefanova and Risch 2013b). Therefore, it is very difficult, or even impossible, to automatically decide whether the triple <4, rdfs:type,: Dataset> is associated with the triple <English, rdfs:type,: Language> or <Greek, rdfs:type,: Language> when reversing. This state of affair poses the issue of how to combine the values of the above four columns for building back the rows of the original CSV. In other words, how do we interrelate the cell values of columns? Concretely, how should we know whether 5 is related to Greek or English, when rebuilding the first row of the CSV source. The issue extends to the values of the other columns as well.

**Figure 3.**
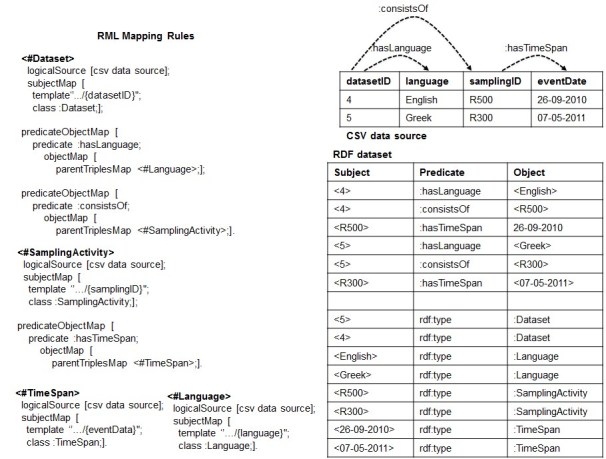
Making explicit potential associations (guided by the target schema MarineTLO (Tzitzikas et al. 2013)) between the columns of the CSV data source.

We noticed that the root of this problem may lie in the fact that potential relationships between columns in the CSV data source are not expressed at the conceptual level through the mapping rules. As shown in Fig. 3, making such associations explicitly (see RML mapping rules of Fig. 3) would produce additional triples that materialize the links among the values of the same row. Specifically, we would know that 4 is connected to English and not with Greek through the triples <4: hasLanguage English>. Likewise, 4 is associated with R500 through the triple <4: consistsOf R500>, which in turn, is related to 26-09-2010 through the triple <R500: hasTimeSpan 26-09-2010>.

**Figure 4.**
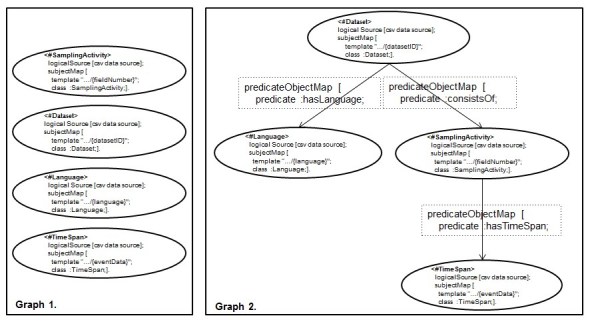
The graph structures underlying the RML rules of Figs 1, 3.

Based on such observation, we asked how we can make sure that we deal with types of scenario exemplified in Fig. 3 for which we would produce the expected result, and not with ones of Fig. 1. To achieve this, we analyzed the structure underlying the RML mapping rules for both cases. In particular, we can schematize such a dependency as a *direct graph* where the *vertices* are the Subjects' part of each rule and the *edges* are their PredicateObjectMaps' part. Fig. 4 shows the two graphs, Graph 1 and Graph 2, obtained from the rules of the Figs 1, 3, respectively. As a result, we observed that the RML rules of Fig. 1, without formalizing the relationships between columns, form a graph with no edges (see Fig. 4, Graph 1) and, the rules of Fig. 3, that they do, form a directed graph forming an n-ary tree (see Fig. 4, Graph 2). Thus, in this paper we make a specific assumption on the graph structure underlying the mapping rules. It is expressed by the following *Dependency Tree Assumption*:

**Dependency Tree Assumption (DTA).** *Given a set of RML mapping rules, S = {tm_1_,.. . , tm_*n*_}, that was used to expose a CSV data source, C, as an RDF dataset D. We use S over D to obtain back C if and only if the directed graph, G, underlying S is one n-ary tree.*

Informally, G will have (a) only one vertice, *root*, that does not have incoming edges, (b) one or more vertices, *leaves*, that do not have outgoing edges, (c) there is at most one path (always starting from the *root* node) that connects two nodes and (d) each node has no more than n children.

**Figure 5.**
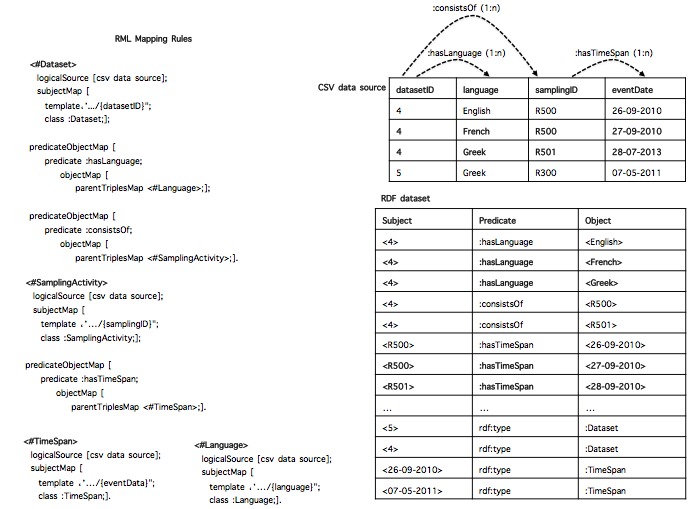
Extention of the example of Fig. 3 with 1:n association cardinality.

***2. Implicit Cardinality Restrictions (ICR).*** It is related to the cardinality of the association between CSV columns. For the sake of clarification, let's consider the example of Fig. 5. The CSV data source contains a number of rows that share the same values, making the relationships: consistsOf,: hasLanguage and: hasTimeSpan of cardinality 1:n. Under such a circumstance we face the issue of multiples range values for the same domain value. For example, <4> has two range values, <R500> and <R501>, through the predicate: consistsOf, and <R500> has also two associated values, <26-09-2010> and <27-09-2010>, through the predicate: hasTimeSpan. When reversing, it becomes problematic to decide which one of the pairs <R500, 26-09-2010> or <R500, 27-09-2010> should be used with the pair <4, French> in order to rebuild the row 2. Likewise for the reconstruction of the row 1. Currently, RDF Data Model does not provide the equivalent concept of "row" for keeping together RDF triples that refer to subparts of the same row (Stefanova and Risch 2013a), expect the notion of "reification" that can be used to support descriptions of a triple or set of triples (Grewe 2010). But it is currently not supported by [R2]RML. For the time being, to copy with such a complexity we make a specific assumption on the instance level of the original CSV data source, expressed as follows:

**Implicit Cardinality Restrictions (ICR)**. *Given an RDF dataset D, we assume that D was generated from an original CSV data source C with associations between columns with only 0:0 or 1:1 cardinality constraints.*

An example of CSV data source that satisfies the ICR assumption is showed in Fig. 3. We will proceed with the design of the RML2CSV algorithm assuming that both the DTA and ICR are satisfied.

### Design description

Once the DTA and ICR are satisfied, the set of RML rules contains all the required information to rebuild the content, row by row, header included. In particular, each rule provides details such as the SubjectMap and PredicateObjectMap that connects two rules (e.g the predicate: consistsOf connects <#Dataset> with <#SamplingActivity>). Taking advantage of such structures, one way to build back a specific row is to exploit the set of rules from the most generic one to the most specific ones. Using a tree nomenclature, it means to visit the n-ary tree from the root to the leaves. We repeat this step for all the values that are instances of the root SubjectMap's Class. To exemplify the main idea, let us consider the RDF dataset and the set of rules of Fig. 3. It contains <#Dataset> as the most generic rule, whom SubjectMap's class is: Dataset and <#TimeSpan> and <#Language> as the most specific ones. The instances of: Dataset are <4> and <5>. Starting with <4>, we have that <4> is related to <English> through the predicate: hasLanguage (see the RDF triple <4,: hasLanguage, English> in the RDF dataset) and to <R500> through the predicate: consistsOf (see the RDF triple <4,: consistsOf, R500> in the RDF dataset), which, in turn, is related to <26-09-2010> through the predicate: hasTimeSpan (see the RDF triple <R500,: hasTimeSpan, 26-09-2010> in the RDF dataset). Organizing such values according to the structural information provided by the RML rules we build a row putting together the associated values, e.g. <datasetID@4, language@English, samplingID@R500, eventDate@26-09-2010>. Likewise for the value <5> we obtain the info of the second row: <datasetID@5, language@Greek, samplingID@R300, eventDate@07-05-2011>. As a result, we have all the required information to rebuild the CSV data source of Fig. 3.

**Algorithm**

To compute automatically such a process we devised a *generic* (as it provides the main steps that can be used also for other types of sources such as XML, JSON, DB) and *extendible* (as it provides the main logic for covering other RML language features as well as) algorithm, RML2CSV, as detailed in Algorithm 1. In particular, line 3 identifies the most generic triple map (it is the one that does not have any incoming edge) and line 4 retrieves the instances of the SubjectMap class of that triple map by using the *SelectDistinctSubejct(classURI, d)* function. The latter is based on the SPARQL query saves as *PREFIX rdf: <http://www.w3.org/1999/02/22-rdf-syntax-ns#> Select distinct ?subject Where { ?subject rdf:type classURI }* executed over the RDF dataset. Finally, we use the set of RML rules to reconstruct all the rows (from line 5 to line 9) using the *ReverseRow* sub-call as reported in the Appendix. Once all the rows are reconstructed, line 10 exports and save them as csv file.

**Algorithm 1.** Reversing an RDF Dataset through the use of RML mapping rules.

INPUT: 1) a set of RML mapping rules S 2) an RDF Dataset d.

OUTPUT: 1) a CSV file.

1: procedure RML2CSV (S, d)

2: reversedCSV[] ← empty; //List of reversed rows.

3: dT ← IdentifyTheMostGenericRMLrule(S); //dT ← the root node. 

4: distinctSubjects[] ← SelectDistinctSubject(dt.getClassURI(), d); 

5: for each subji in distinctSubjects[] do

6: partRevRowi [] ← empty; //List of rowItem.

7: currPred ← empty; //a predicate of an RDF triple.

8: reversedRowi[] ← ReverseRow(S, subji , partRevRowi[], currPred, dT, d);

9: reversedCSV[].add(ReversedRowi[]);

10: Export reversedCSV[] as a csv text file.

### Funding

The work has been supported by the LifeWatchGreece project, funded by GSRT, ESFRI Research Infrastructures, Structural Funds, OPCE II (Act Code: 384676).

## Web location (URIs)

Download page: https://bitbucket.org/carloallocca/rml2csv

## Technical specification

Programming language: Java

Operational system: Windows or Linux or Mac

Interface language: Java

## Repository

Type: Git

Browse URI: https://bitbucket.org/carloallocca/rml2csv

Module: packages gr.hcmr.imbbc.rmlreverse.

## Usage rights

### Use license

Creative Commons CCZero

## Implementation

### Implements specification

We have implemented RML2CSV on top of RML, based on the fact that, in comparison to the other approaches, it provides a uniform way to access different types of data sources such as CSV, XML, JSON and DB. Consequently, we believe that enabling the corresponding reverse processes within the same framework it would not only strengthen the latter but also make it to be used by a much larger community, as well as to extend it to support other type of data source, beyond CSV. The current implementation of the RML2CSV can be found at https://bitbucket.org/carloallocca/rml2csv (see the three packages). It is important to highlight that the extension from RML Lite to RML does not have any logical implication on the presented algorithm. Moreover, we are currently working on it in order to cover the entire RML Mapping Language.

## Additional information

### Evaluation and Results

The general goal of evaluating RML2CSV is to answer the following (related) questions: 1. Does it solve the problem that is supposed to? 2. Does it work correctly under all the assumptions? To answer such questions, we designed a set of content based criteria to estimate the extent to which the reversed data source (csv^r^) overlaps, row by row, with the original one (csv^o^). To this end, we based such a comparison on computing a similarity measure between csv^r^ and csv^o^, as expressed in the following:

\begin{varwidth}{50in}
        \begin{equation*}
            ContentSimilarity(csv^{r}, csv^{o}) = 1 - contentDistance(csv^{r}, csv^{o}) \hspace{10mm}(1)
        \end{equation*}
    \end{varwidth}

where the contentDistance intends to measure the number of rows and the extent to which they contain the same information. It is defined as in the following:

\begin{varwidth}{50in}
        \begin{equation*}
            contentDistance(csv^{r}, csv^{o})= \sum\limits_{i=1}^m \frac{1}{m} \times rowDistance(row_{i}^{o}, row_{i}^{r}) \hspace{10mm}(2)
        \end{equation*}
    \end{varwidth}

where m is the number of rows of the csv^o^, row_i_^r ^is computed by CorrRow(row_i_^o^) which is a function to calculate the corresponding i-th row in the reversed CSV and, the rowDistance measures the number of cells and the extent to which they contain the same values. It is defined as in the following:

\begin{varwidth}{50in}
        \begin{equation*}
            rowDistance(row_{i}^{o}, row_{i}^{r}) = \sum\limits_{i=1}^n \frac{1}{n} \times cellDistance(cell_{i}^{o}, cell_{i}^{r}) \hspace{17mm}(3)
        \end{equation*}
    \end{varwidth}

where n is the length of row_i_^o ^, cells_i_^r^ is computed by CellRow(cell_i_^o^) which is a function to calculate the corresponding i-th cell in the reversed CSV and the *cellDistance* is based on a string compare function checking whether the reversed value is the same as of the original one. Thus, *cellDistance = 1*, it means that the two values are different whereas *cellDistance = 0* means that the two value are syntactically equal.

Combining (1), (2) and (3) together we have that: if (3) is always equal to 0, meaning that anytime we compare two rows they always contain the same values, then (2) is equal 0, meaning that csv^r^ and csv^o^ contain the same content. In this case, (1) would measure a similarity equal to 1. On the contrary, if (3) is always equal to 1, meaning that anytime we compare two rows they always contain different values, then (2) is equal 1, meaning that csv^r^ and csv^o^ contain different content. In this case, (1) would measure a similarity equal to 0. To face with the

**Figure 6.**
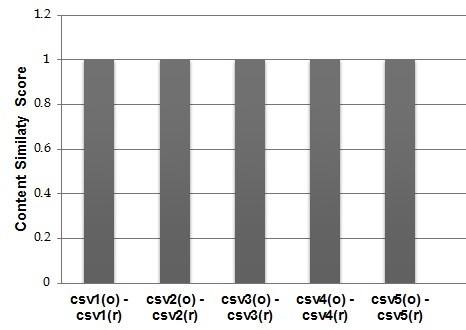
The results of comparing csv ^o^ with csv ^r ^ (Suppl. material 1).

The current evaluation is based on a collection of five CSV data sources from Biodiversity domain, containing mainly occurrence data from the MedOBIS (Biogeographic information system for the eastern Mediterranean and Black Sea (Arvanitidis et al. 2006)). They are characterized by a different column-based structure containing from 4 to 12 columns (e.g. datasetID, language, fieldNumber, different types of measurements just to report a few). Before transforming them into RDF datasets we applied a pre-preprocessing to make sure that their content would not generate any of the issues analyzed in the *Study Area Description* section and further analyzed in the *Discussion* section. After running RML2CSV we compared csv^r^ and csv^o^ according to the criteria (1), (2) and (3). The results are shown in Fig. 6.

As it can be noticed, RML2CSV reconstructed all the five CSVs with a content up to 100% overlapped with the original ones. This very initial evaluation does not pretend to demonstrate the correctness or completeness of proposed approach, but it posed the base and encourage us for a thorough evaluation of the RML2CSV efficiency and effectiveness.

### Discussion

We designed and implemented our algorithm, RML2CSV, taking into account the DTA and the ICR assumptions. Now, we discuss how to build upon the current achievemnts in order to suggest solutions for relaxing the two assumptions.

**Figure 7.**
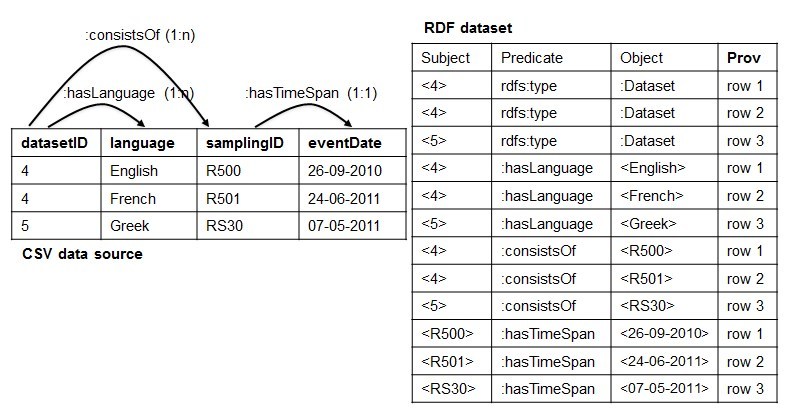
Exposing a CSV data source with 1:n implicit constraints using RML rules Fig. 3.

**More about the DTA and ICR:** Being aware that they could be too limited for dealing with a wide range of real cases, we propose two solutions for relaxing the two assumptions. The first is based on extending the forward process producing an auxiliary structure for keeping links between RDF triples that refer to the subparts of the same row. Fig. 7 shows an approach based on a quad (instead of triple) structure where the fourth component describes the row that the triples are connected to. This would mean to change the workflow of the entire forward process of RML. The second, that is the one we consider in the next developments, is based on the only and more realistic assumption that the CSV data source should have a structure containing at least one column with unique value that could be used as *key.* Based on this, the RML rules could be extended with an appropriate and domain independent relationship for keeping links between RDF triples that refer to the subparts of the same row, through the use of such ''key column''. Fig. 9 shows an example where *rowNumber* exemplifies the role of a key-column of the CSV data source. And, in order to keep the link between the different values of the same row we extended each RML rule with an PredicateObjectMap that uses the predicate: hasIdentifier which, in turn, refers to <\#RowNumber> rule. By doing so, the forward process generates triples such as <4, rdf:type,: Dataset>, <4,: hasIdentifier, 287>, <$English,: hasIdentifier, 287> and <ka\_la\_2002,: hasIdentifier, 287> that will indeed support the reverse process to distinguish the values of the row 287 from those of the row 285. In this way the issue of having associations of cardinality 1:n, or more in general m:n, would be solved.

**Figure 8.**
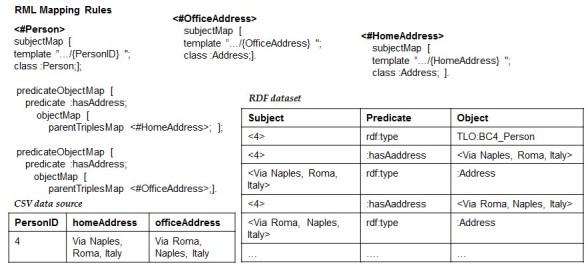
An example of a very “low” quality mappings.

**Figure 9.**
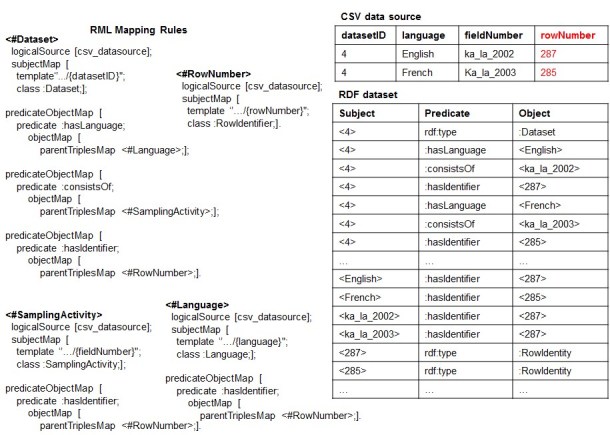
An example of a key value column.

​**Mapping Quality Level:** Fig. 8 shows a case of interpreting two different columns, homeAddress and officeAddress, with the same predicate: hasAddress whom parentTripleMap refers to two different RML rules which, in turn, have the same URI resource for the corresponding SubjectMap class. In this case, it would be difficult to decide the home and the office address of <4> when reversing, as both <Via Naples, Roma, Italy> and <Via Roma, Naples, Italy> are related to <4> through the same predicate: hasAddress. Another similar example could be that not all the data of the original CSV are mapped or heavy post-processing (e.g. transforming latitude, longitude and coordinateUncertaintyInMeters in a polygon) that can’t be inverted is applied. Such type of issues could be related, in one way or another, to the quality of mapping rules. In other words, a very “*high* ”, in quality, mapping rule set would avoid the above issues, whereas a very “*low* ”, in quality, mapping rule set would generate not only the one discussed above but also others that have not been considered yet. As we are moving out of the scope of the current work, for such type of issues we will rely on the mapping quality outcomes (Dimou et al. 2014), setting new principles and guidelines for supporting the user on how and what to expose and, at the same time, delivering mapping services for checking the quality.

### Related Work

To the best of our knowledge, there is no other study investigating the reversing of an RDF dataset for reconstructing the original tabular data source of CSV type. On the contrary, several solutions exist to execute mappings from different types of data sources and serialisations to the RDF data model. The R2RML W3C recommendations (Das et al. 2012) and its direct extension RML (Dimou et al. 2013) are the two main approaches to expose different types of data sources as an RDF dataset. X3ML (Kondylakis et al. 2006) implements a similar declarative approach by providing an XML-based mapping language for consuming XML records and producing RDF in various serializations. Moreover, mapping languages were defined to support mapping for tabular data in CSV and spreadsheets to RDF. They include the XLWrap’s mapping language (Langegger and Wöß 2009) that converts data in various spreadsheets to RDF, the declarative OWL-centric mapping language Mapping Masters M2 (O’Connor et al. 2010) that converts data from spreadsheets into the Web Ontology Language (OWL), and Vertere (https://github.com/knudmoeller/Vertere-RDF). Furthermore, there were other solutions proposed for mappings of data in tabular structure to the RDF data model but in those solutions the mapping solutions are tiedto the implementation (a complete list can be found at http://www.w3.org/wiki/ConverterToRdf). Overall, none of these solutions considered the reverse mapping, namely from the generated RDF dataset to the original tabular data source. Except for a few cases dealing with rebuilding Database schema and instances from RDF documents).

In particular, RDF2RDB (Teswanich and Chittayasothorn 2007)provides an approach to transform an RDF document into RDB. Under the assumption that an RDF document - not defined in the corresponding paper- is equivalent to the RDF dataset as used in this paper, one could think of using RDF2RDB for reconstructing the CSV file by storing the RDF dataset in one RDB table and then export it as CSV file. Unfortunately, the approach does not work to achieve the reconstruction of the CSV data source according to the original structure. Let us explain why: The use of RML mapping rules to transform a CSV data source in RDF dataset is based on a target schema or vocabulary that is chosen by the mapping creator. One of the consequences of that is that the original CSV structure may be completely destroyed (e.g. eventData become TimeSpan, see Fig 1.). Therefore, the application of RDF2RDB would produce a single table, ready to be exported as CSV, that reflects the used target schema or vocabulary. In contrast, our work aimed at rebuilding the CSV according to its original structure.

Similarly (Stefanova and Risch 2013a) and (Stefanova and Risch 2013b) present SAQ- Semantic Archive and Query - a system for recreating of relational database archived as RDF dataset. Once again, one could consider to use SAQ for performing the forward and the backward transactions. Accordingly, there is very high price to pay which is based on the fact that the RDB to RDF mapping is based on the direct mapping (Arenas et al. 2012) where the structure of the result RDF dataset directly reflects the RDB schema elements. In other words, There is a direct correspondence between the target RDF vocabulary and the names of the RDB schema elements. In contrast, RML2CSV is built on top of RML that provides a mapping language for expressing customized mappings from a heterogeneous data source to an RDF data model that does not necessary reflect the original structure as showed above. R2D (Ramanujam et al. 2009a, Ramanujam et al. 2009b) generates a relational view through the execution of SQL queries over an RDF store. Similarly to Jena (HPCompany and F. 2002) through the use of the service TupleQueryResultFormat allowing to format the query results in a number of ways, including CSV, JSON and so on. But they don't do it according to the original schema. Finally, (Terwilliger et al. 2008) and (Drew 2014) face the problem of converting XML, and not RDF, Schema to relation tables.

Unfortunately, all these existing approaches are rather limited for our scenario either because they do not consider the reverse problem at all or because they face it in different context and targetting diverse goal. While they contribute interesting elements for us to build on, we focus here on how to perform the reverse process for the case of column-based structured data source of CSV type w.r.t its original data structure and not any. Furthermore, as our solution is based on [R2]RML mapping language, it provides the additional advantage that we can perform both transactions, CSV data source to RDF dataset and vice-versa, within the same framework, that none of the discussed work does.

### Conclusion and Outlook

In this paper we argue that an important aspect of long-term preservation of digital objects, such RDF datasets, is to provide full support for reusing such data, including mechanisms to bring back the data to their original format. To achieve this, in this work we investigated on how to perform the reverse process for the case of column-based data source such as tabular data. In particular, we devised an algorithm, the RML2CSV, for transforming an RDF dataset into its original data structure, through the use of the same RML mapping rules used to generate the set of RDF triples. The results of the evaluation showed that RML2CSV rebuilds the same data content with the same data structure under certain assumptions.

In the future, a thorough evaluation of RML2CSV efficiency will be performed. In addition, we have planned to extend RML2CSV to dealwith any type of constraints between columns (e.g. 1:n and, more general m:m) as discussed in *Discussion* section and to cover all RML mapping languages. As a long term objective, we plan to design and implement the back transformation to any type of relevant formats including XML, JSON and DB, by taking advantage of the achievements presented in this paper.

**Appendix.**

Algorithm 2 Reversing a single CSV row from an RDF Dataset through the use of RML mappings.

1: procedure ReverseRow(subji, partRevRowi [], currP red, dT, d)

2: currentRowContent[] ← partRevRowi [];

3: if dT = null then

4: currSubjectValue ← subji;

5: if dT.PredicateObjectMaps[] = null ∧ dT is not the root node then

6: return currentRowContent[];

7: if dT.PredicateObjectMaps[] = null ∧ dT is the root node then

8: termName ← dT.SubjectMap.getTemplate.localName; 

 . we need to check if SubjectMap has a template

9: rowItem ← TermName@currSubjectValue;

10: currentRowContent[].add(rowItem);

11: return currentRowContent[];

12: else

13: for i = 1 to dT .P redicateObjectM aps[].length do

14: currP red ← dT.PredicateObjectMap[i].getPredicate;

15: objectMap ← dT.PredicateObjectMap[i].getObjectMap;

16: if objectMap contains a parentTripleMap then

17: parentTriplesMap ← objectMap.getParentTriplesMap();

18: tripleMapName ← parentTriplesMap.getName();

19: nextTripleMap ← Search(dT, tripleMapName);

20: termName ← nextTripleMap.SubjectMap.getTemplate.localName; 

 . we need to check if SubjectMap has a template

21: className ← nextNodeToExplore.SubjectMap.getClass;

22: nextSubject ← SelectDistinctObject(d, currSubjectValue, currPred, className); 

 . SPARQL query where (currSubjectValue, currPred, ?object) and (?object rdf:type className)

23: cellItem ← termName@nextSubject;

24: currentRowContent[].add(cellItem);

25: ReverseRow(nextSubject, currentRowContent[], currPred, nextTripleMap, d);

26: if dT is the root then

27: termName ← dT.SubjectMap.getTemplate.localName;

28: rowItem ← termName@subji;

29: currentRowContent[].add(rowItem); 

 . if it is not already added

30: if objectMap contains a rr:reference then

31: print(”Not detailed for space reason.”);

32: return currentRowContent[];

33: else return null;

## Supplementary Material

Supplementary material 1 RML2CSV evaluationn Data type: RML2CSV evaluation data
File: oo_45207.xlsx Carlo Allocca
